# Chimeric antibodies with extended half-life in ferrets

**DOI:** 10.1111/irv.12273

**Published:** 2014-08-27

**Authors:** Thomas C Nesspor, Bernard Scallon

**Affiliations:** Janssen Research and Development, LLCSpring House, PA, USA

**Keywords:** Ferret, half-life, immunogenicity, influenza, pharmacokinetics

## Abstract

**Background:**

Ferrets have long been used as a disease model for the study of influenza vaccines, but a more recent use has been for the study of human monoclonal antibodies directed against influenza viruses. Published data suggest that human antibodies are cleared unusually quickly from the ferret and that immune responses may be partially responsible. This immunogenicity increases variability within groups and may present an obstacle to long-term studies.

**Objective:**

Our aim was to identify an antibody design with reduced immunogenicity and longer circulating half-life in ferrets.

**Methods:**

The constant region coding sequences for ferret immunoglobulin G were cloned, and chimeric human/ferret antibodies were expressed and purified. Some of the chimeric antibodies included substitutions that have been shown to extend the half-life of human IgG antibodies. These chimeric antibodies were tested for binding to recombinant ferret FcRn receptor and then evaluated in pharmacokinetic studies in ferrets.

**Results:**

A one-residue substitution in the ferret Fc domain, S252Y, was identified that increased binding affinity to the ferret neonatal receptor by 24-fold and extended half-life from 65 ± 27 to 206 ± 28 hours or ∼9 days. Ferrets dosed twice with this surrogate antibody showed no indications of an immune response.

**Conclusion:**

Expressing the variable region of a candidate human therapeutic antibody with ferret constant regions containing the S252Y substitution can offer long half-life and limit immunogenicity.

## Introduction

Ferrets are susceptible to infection with human influenza virus and show symptoms that mimic those seen in humans.^1^,^2^ For this reason, ferrets have routinely been used to study the effects of influenza vaccines. However, a more recent application has been the study of human monoclonal antibodies (mAbs) directed against influenza viruses.^3^ Such studies have revealed the unusually short half-life of human mAbs in ferrets and hinted that immune responses may have driven clearance at later time points.^4^

A long-used strategy to minimize immune responses in animal models has been to replace the constant region sequences of an antibody with the corresponding constant regions from the animal species to be used for *in vivo* studies.^5^ Even though ∼30% of the sequences in such a chimeric mAb are derived from the original mAb, the incidence of immune response can be substantially reduced.^6^ In addition, the half-life of the chimeric mAb may be extended by optimizing binding to the neonatal Fc receptor (FcRn). Antibodies owe their long half-lives to recycling through endosomes and release back into the extracellular space. FcRn binds antibodies in the endosome at low pH and routes them to the cell membrane where they are released at neutral pH.^7^ Substitutions that improve binding to the FcRn at low pH and extend the half-life of human mAbs have been extensively studied.^8^ For instance, position 252 has been known to be important for FcRn interactions and substitution of the methionine at this position with a tyrosine (M252Y) in human mAbs has been shown to increase affinity for human FcRn.^9^ Moreover, when the M252Y substitution was combined with two additional substitutions, S254T and T256E (to make the YTE triple mutant), binding to human and cynomolgous monkey FcRn at pH 6·0 was increased 10-fold and half-life in monkeys was increased by more than threefold.^10^ Another pair of substitutions, M428L and N434S, has also been shown to increase the half-life of human mAb in monkeys by threefold.^11^ These observations suggest that a similar strategy, and perhaps the same substitutions, could be used to prolong half-life of antibodies in ferrets.

## Methods

### Cloning of DNA encoding ferret immunoglobulin constant sequences and FcRn

RNA was isolated from ferret kidney, lung, liver, and spleen using Trizol reagent (Life Technologies, Grand Island, NY, USA) and the RNeasy kit (Qiagen, Germantown, MD, USA). The RNA was then reverse-transcribed using an oligo(dT) primer and the Superscript III First Strand Synthesis System (Life Technologies). The cDNA product was amplified by polymerase chain reaction (PCR) with primers designed for the ferret immunoglobulin G (IgG) heavy chain (HC) constant region, kappa light chain (LC) constant region, FcRn alpha chain, or β_2-_microglobulin (β2m). The forward and reverse primer sequences according to the nomenclature of the International Union of Pure and Applied Chemistry were 5′-GGTCACCGTGTCCTCAGC-3′ and 5′-GCGTGCGGCTCATTTACC-3′ for the HC, 5′-AAGGTGGAAATCAAACGG-3′ and 5′-ATAGGTGGTGGGTGCTGC-3′ for the LC, 5′-ATGSGGVKYCCBCGGCCTC-3′ and 5′-TTCCGATCACGGGCACGG-3′ for the FcRn, and 5′-CTACTCCGGTGGCGATGG-3′ and 5′-AAACCTCCATGATGCTGG-3′ for β2m. Selected reactions underwent a second round of PCR amplification using nested primers which included restriction sites to allow cloning of the PCR products. The forward and reverse primer sequences were 5′-TTTCGTACGGCTTCCACCACGGCCCC-3′ and 5′-AAATGATCATCATTTACCCGGAGACTGG-3′ for the HC, 5′-TTTCGTACGAATGATGCCCAGCCATCCG-3′, and 5′-AAATGATCACTAGGCCACTCATTGGCAC-3′ for the LC, 5′-TTTAAGCTTAGGTGCGTCCTTCGAGCCACCATGSGGVKYCCBCGGCCTC-3′ and 5′-TTTTCTAGAGGAGGATCTGGCTGGTG-3′ for the FcRN, and 5′-TTTAAGCTTGCCACCATGGCGCTTCTCTGGACG-3′ and 5′-AAATCTAGATTAGTTGTCTCGCTCCC-3′ for β2m. The PCR products were cloned into mammalian expression vectors under control of the CMV promoter. The plasmid for expressing ferret FcRn also included coding sequence for a 6× His tag downstream of the cloning site.

### Preparation of chimeric antibodies and variants

The HC and LC variable regions from a humanized mAb specific for the F glycoprotein of respiratory syncytial virus (RSV) were chosen for incorporation into the test antibodies used in these studies, as no Fab-mediated binding to ferret tissues would be expected. These variable regions were cloned into their respective expression plasmids containing either the ferret IgG HC constant regions or kappa LC constant regions. The chimeric HC expression plasmid was then mutated using the QuikChange Kit (Agilent, Santa Clara, CA, USA) to generate variants with substitutions intended to extend half-life. The mutagenesis primer sequences were 5′-CCCAAGGACACCCTCATGATTTCCCGAACCCCC-3′ and 5′-GGGGGTTCGGGAAATCATGAGGGTGTCCTTGGG-3′ for the S252M variant, 5′-CCAAGGACACCCTCTACATTTCCCGAACCC-3′ and 5′-GGGTTCGGGAAATGTAGAGGGTGTCCTTGG-3′ for the S252Y variant, 5′-CCCAAGGACACCCTCTACATTACCCGAGAGCCCGAGGTTACATGC-3′ and 5′-GCATGTAACCTCGGGCTCTCGGGTAATGTAGAGGGTGTCCTTGGG-3′ for the YTE variant, and 5′-GAAGCCCTACACAGCCATCACACGCAG-3′ and 5′-CTGCGTGTGATGGCTGTGTAGGGCTTC-3′ for the N434S variant. The chimeric antibodies were expressed in HEK 293F cells and purified using an AKTA purification system (GE Healthcare, Piscataway, NJ, USA). For this, both a HiTrap protein G HP column and HiTrap MabSelect Sure protein A column (both from GE Healthcare) were used in series. The purified products were analyzed by SDS-PAGE gels using pre-poured 4–12% Bis-Tris gels and MOPS running buffer (Life Technologies) as well as size exclusion high-performance liquid chromatography (SE-HPLC) using a TSKgel G3000SWx1 column (TOSOH Biosciences, King of Prussia, PA, USA) on a Waters Alliance HPLC system.

### Preparation of recombinant soluble ferret FcRn

The plasmids encoding the extracellular portion of the α chain and β2 microglobulin were co-transfected into HEK293E cells. The His-tagged ferret FcRn α chain/β2 microglobulin complex secreted by the cells was purified from the culture media using a 5-ml HisTrap HP column (GE Healthcare) on an AKTA Purifier 100 followed by a 320-ml HiLoad 26/60 Superdex 200 size exclusion column (GE Healthcare) on an AKTA FPLC system. The purity was assessed by SDS-PAGE and SE-HPLC. SDS-PAGE was performed using NuPAGE 4–12% Bis-Tris gels with NuPAGE MES SDS running buffer (Life Technologies) under both reducing and non-reducing conditions. SE-HPLC was performed on a Waters 2695 HPLC instrument using a TSKgel G3SW_XL_ PEEK column (TOSOH Biosciences).

### Ferret FcRn binding assays

Using an AlphaScreen-based protein–protein interaction assay (Perkin Elmer, Waltham, MA, USA), chimeric antibodies were assayed for ferret FcRn binding in a competitive binding format. Titrated amounts of chimeric mAb were added to biotinylated wild-type (WT) chimeric mAb (1–2 μg/ml final), His-tagged ferret FcRn (4–8 μg/ml final), a 1:50 dilution of nickel-coated AlphaScreen acceptor beads, and a 1:50 dilution of streptavidin-coated AlphaScreen donor beads in a white polystyrene half-area flat bottom assay plate (Corning, Tewksbury, MA, USA). The plate was incubated for 1 hour at RT in the dark, and then the amount of luminescence triggered by the proximity-dependent transfer of singlet O_2_ was measured using an Envision plate reader.

### RSV F glycoprotein binding assays

Binding to RSV F glycoprotein was measured in a competitive binding assay. The first step was the capture of 1 μg/ml RSV F glycoprotein on a copper-coated plate form Thermo Scientific via its His tag. Test antibodies were then titrated in assay buffer (PBS + 10% ChemiBLOCKER; EMD Millipore, Billerica, MA, USA) containing 0·5 μg/ml biotinylated human anti-RSV F glycoprotein antibody. The mixture was added to the plate and incubated for 1 hour. StreptAvidin-HRP (Jackson Immunoresearch) at a 1:10 000 dilution was used to detect bound biotinylated mAb.

### Pharmacokinetic studies in ferrets

All animal studies were performed at Covance Laboratories, Inc. (Madison, WI, USA) using male ferrets at least 16 weeks old and weighing between 1 and 2 kg. For both studies, a vascular access port was surgically inserted into the jugular vein 5 days prior to dose administration. At the start of the study, the ferrets received a single 2 mg/kg dose of test mAb administered intravenously via the vascular access port. Blood samples were collected into tubes containing sodium heparin anticoagulant and kept on ice until centrifugation to isolate plasma. To quantitate the amount of test mAb in plasma samples, Streptavidin Gold MultiArray plates (Meso Scale Discovery, Rockville, MD, USA) were blocked and a biotinylated mouse anti-idiotype mAb specific for the test antibodies, diluted to 1 μg/ml, was captured on the plates. After washing, ferret plasma dilutions were added and the plate incubated for 1 hour. Following the incubation, the plates were washed and the detection reagent added. The ruthenium-labeled F(ab′)2 fragment of a donkey anti-human IgG(H + L) mAb (Jackson ImmunoResearch, West Grove, PA, USA) at 0·25 μg/ml was used for detecting human mAb. Chimeric mAb was detected using ruthenium-labeled anti-ferret IgG (Rockland Immunochemicals, Gilbertsville, PA, USA), which had been cross-absorbed against human mAb before use. This reagent was diluted in 20% mouse serum to block cross-species reactivity. After 1 hour incubation, the plates were again washed, and MSD read buffer (1×) added to each well. A Sector Imager 6000 (Meso Scale Discovery) was used to measure the electrochemiluminescent signal resulting from bound test mAb, and the amount of mAb was quantitated by comparison with concentration standards. Assay sensitivity was not affected by ferret plasma amounts up to 20% and was quantitative at concentrations of mAb between 0·1 and 100 ng/ml, representing a four-log linear range. Terminal half-lives of test antibodies were calculated using a two-phase decay nonlinear regression model in the GraphPad Prism software package (GraphPad Software Inc., La Jolla, CA, USA). Outliers were identified using the Grubbs test with a *P*-value of >0·05.

### Measuring ferret immune responses to test antibodies

Ferret anti-human mAb antibodies were detected in a bridging assay that entailed coating human mAb on plates, adding titrating amounts of ferret plasma sample, and detecting bound ferret antibodies with the same human mAb, this time labeled with ruthenium. Biotinylated test mAb at 1 μg/ml was captured on blocked Streptavidin Gold MultiArray plates. Diluted plasma samples were then incubated on the plate for one hour after which the plates were washed and ruthenium-labeled test mAb at 1 μg/ml was added. After 1 hour incubation, the plates were washed, MSD read buffer was added, and the amount of bound mAb detected as before.

## Results

### Pharmacokinetics and immune responses after a single dose of human mAb

**Figure 1:**
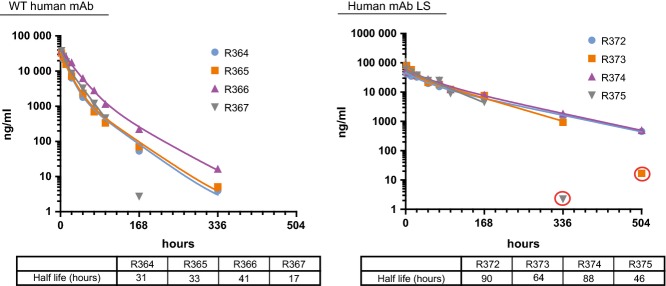
Human mAb plasma concentrations over time. mAb concentrations in the plasma of individual ferrets (identified by R numbers) dosed with wild-type (WT) human mAb (left) or an LS mutant (right) are shown. Terminal half-life values are given a table below each graph. Outliers are circled in red.

**Figure 2:**
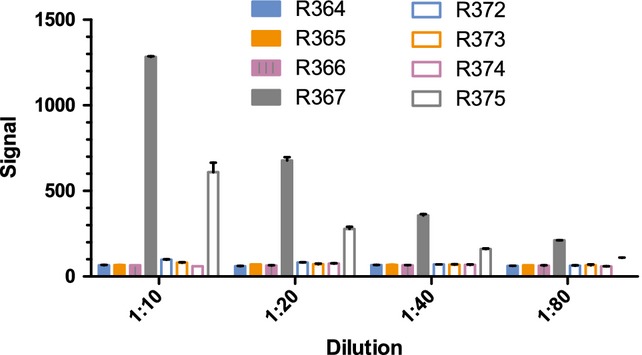
Immune response. Bars represent the relative amounts of ferret anti-human mAb antibodies present in plasma samples from ferrets given either wild-type (WT) human mAb (R364-R367) or an LS mutant (R372-R375) taken 504 hours after injection.

Our initial efforts were focused on determining the PK of a human IgG antibody in ferrets and testing for the induction of polyclonal ferret anti-human IgG antibodies (commonly referred to as antidrug antibodies or ADA). For this study, male ferrets were intravenously injected with a single 2 mg/kg dose of a human IgG1 mAb that was expected to not bind to any ferret antigens (specific for RSV F glycoprotein). Blood samples were then collected 2, 6, 12, 24, 48, 72, 96, 168, 336, and 504 hours after injection, and plasma prepared from each sample. In addition to the WT mAb, a second mAb containing the half-life extending substitutions, M428L and N434S (LS), was also tested. Concentrations of human mAb in the plasma samples were determined using an electrochemiluminescent assay. Of the eight animals in this study, two experienced an acute drop in test mAb concentration resulting in values that were identified as outliers using the Grubbs test (Figure1). Ferret anti-human mAb antibodies were detected in one of the two ferrets, R375, using a bridging assay (Figure2). The negative result observed for the other outlier, R373, may have been due to residual human mAb test article in the plasma sample inhibiting binding of ferret antibodies to the immobilized human mAb on the assay plate. ADA were detected in one additional ferret, R367, which experienced a drop in mAb concentration that did not reach significance (*P* < 0·05) in the Grubbs outlier test. Excluding these three time points, the average half-life of the WT human IgG was 31 ± 10 hours, whereas the half-life of the LS variant averaged 72 ± 21 hours. Despite the extended half-life of the LS variant, the detection of anti-human mAbs prompted preparation of a chimeric mAb with human variable regions and ferret constant regions.

### Cloning of ferret IgG constant region sequences

**Figure 3:**
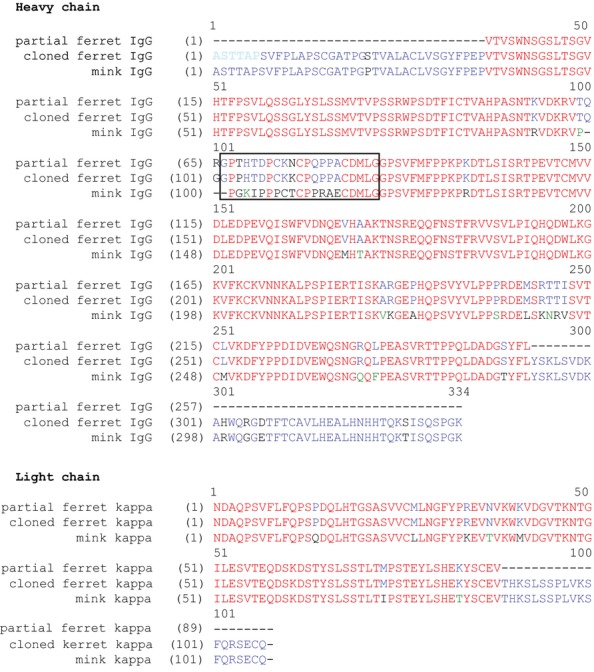
Alignment of cloned ferret IgG heavy and light chain sequences with the previously reported partial ferret IgG sequence and mink sequence. The six amino acids derived from a primer based on the mink sequence are in light blue, and a box denotes the putative hinge region.

Because the amino acid sequences of the ferret IgG constant regions were only partially known, we cloned cDNAs encoding the complete constant regions for the ferret IgG HC and LC. This was done using RNA isolated from ferret kidney, lung, liver, or spleen, and DNA primers designed based on the partial ferret sequence and the mink sequences available in public databases. The ferret DNA sequences were reverse-transcribed, PCR-amplified, and sequenced. Three amino acid sequence differences were found between the consensus ferret HC sequence and the partial database sequence (GenBank Accession Number EZ459103), suggesting that the ferret may have multiple IgG isotypes. All of the differences were in a region that aligned with the hinge region of human IgG1. The ferret HC constant region sequence was 89% identical to the mink sequence with most of the differences again occurring in the hinge region (Figure3). It should be noted that the first six amino acids of the cloned ferret HC sequence were derived from a primer based on the mink sequence, and therefore, the possibility exists that one or more of the first six amino acids do not match the endogenous ferret sequence. The cloned ferret LC sequence was identical to the partial sequence found in the database (GenBank Accession Number EZ457190) and shared 94% identity with the mink sequence.

### Preparation of chimeric human/ferret antibodies

**Figure 4:**
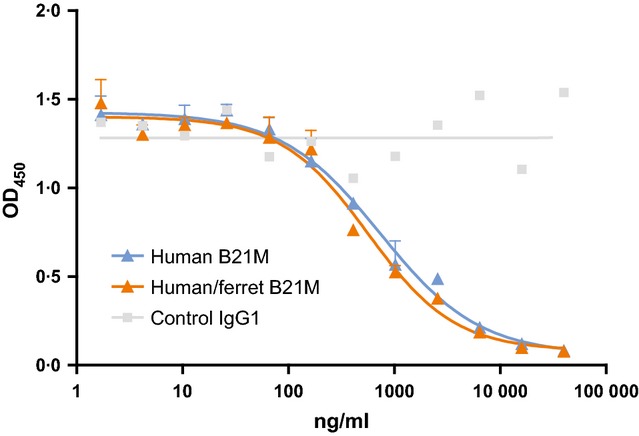
Competition binding to RSV F glycoprotein. A nonlinear equation was used to fit a line to the data points and calculate IC50. RSV, respiratory syncytial virus.

To prepare chimeric mAbs for testing, variable regions from a human anti-RSV mAb were used because they would not be expected to bind endogenous ferret proteins, which could affect pharmacokinetics. Expression plasmids were prepared by splicing the anti-RSV variable regions upstream of the newly cloned ferret IgG constant regions. In addition, amino acid substitutions that have been shown to extend the half-life of human antibodies were introduced into the ferret Fc. The position given for these substitutions is from application of the human antibody EU numbering system to the ferret sequence based on homology. Specifically, variants were made in which the serine at position 252 was replaced with either a methionine (S252M), the amino acid found in human IgG, or a tyrosine (S252Y). A third variant combined the S252Y substitution with two other substitutions: S254T and T256E (YTE) to create a triple mutant. A fourth variant substituted the asparagine at position 428 with serine (N434S). The WT chimeric mAb and the four variants were expressed transiently in HEK293E cells. Protein A and protein G columns aligned in series were used in purification. Initially, protein G was used based on work by Martel and Aasted^12^ who first reported the purification of ferret antibodies, but after ferret mAb was detected in the protein G column flow-through, a protein A column was added to improve capture of the ferret antibodies. The purified antibodies were analyzed by SDS-PAGE under denaturing and both reducing and non-reducing conditions, and the banding patterns were consistent with the expected sizes of the fully assembled mAbs and individual HCs and LCs. SE-HPLC chromatograms showed a single, sharp peak characteristic of a homogenous, monodisperse mAb (data not shown). To access structural integrity, the WT chimeric mAb was tested for binding to its antigen, RSV F glycoprotein, in a competitive ELISA format, and shown to retain full antigen binding (Figure4). The calculated IC50 values were 736 ± 156 and 565 ± 105 ng/ml for the human and chimeric mAbs, respectively, demonstrating that the structure of the human variable regions was functionally intact when expressed with ferret constant regions.

### Cloning and expression of soluble ferret FcRn

FcRn receptor binding is commonly used to evaluate antibodies engineered to have an extended half-life. FcRn is made up of an alpha and a beta chain, yet a search of public databases revealed only the ferret beta chain sequence, also known as β2-microglobulin (β2M). To clone DNA encoding the ferret alpha chain, oligonucleotide primers were designed based on the alignment of known sequences from several species. Using RNA from ferret tissues, PCR amplicons were generated and then sequenced. The consensus sequence shared 92% identity with the dog FcRn alpha chain, the closest relative whose sequence was known. The Fc-binding portion of the cloned alpha chain was then expressed with ferret β2M in HEK293 cells to generate soluble ferret FcRn (sFcRn). Following purification, the purified FcRn was analyzed by SDS-PAGE under reducing and non-reducing conditions (data not shown). Two bands were seen migrating near the expected molecular weight of the alpha chain, 30·1 kDa, and a single band near the expected molecular weight of the beta chain, 11·5 kDa. The doublet may have been due to glycosylation heterogeneity as the ferret alpha chain contains two N-linked glycosylation motifs and multiple glycoforms have been observed with FcRn from other species.^13^

### Chimeric mAb binding to ferret FcRn

**Figure 5:**
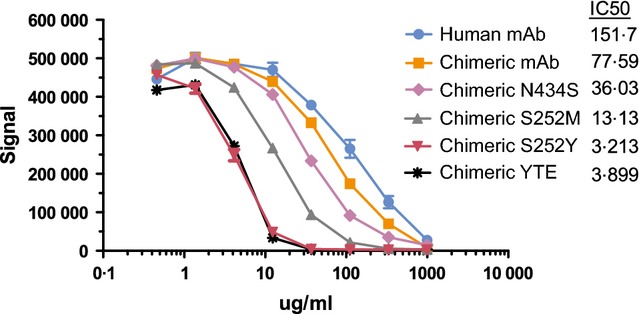
Ferret FcRn binding. The binding of human and chimeric mAbs to ferret FcRn at pH 6·0 was measured in a competition format.

The human/ferret chimeric mAbs were tested for binding to ferret FcRn at pH 6·0 to mimic conditions in the endosomes where IgG-FcRn binding occurs (Figure5). This competitive binding assay used AlphaScreen technology in which the proximity-dependent transfer of singlet oxygen from the donor beads to acceptor beads upon excitation can be detected. For this, WT chimeric mAb was captured on streptavidin-coated donor beads and His-tagged ferret FcRn was captured on nickel-coated acceptor beads. Inhibition of the interaction between the donor and acceptor beads by test mAbs in solution was then measured. The test mAbs were titrated, and the resulting inhibition curves used to calculate IC_50_ values. The IC_50_ values for the human and chimeric mAbs were 152 and 78 μg/ml, respectively. This twofold difference indicated that, even without additional Fc engineering, human/ferret chimeric mAbs might have a longer half-life than human mAbs. All four of the variants gave IC_50_ values that were lower than the WT chimeric mAb, indicating stronger ferret FcRn binding. The largest improvements were seen when the serine at position 252 was mutated. Changing this amino acid to a methionine, as found in the human sequence, resulted in an IC_50_ of 13 μg/ml and mutating it to a tyrosine reduced the IC_50_ to 3·2 μg/ml, a 24-fold improvement over wild type. The YTE variant, which added two mutations in addition to S252Y, did not show additional improvement beyond S252Y by itself.

### Pharmacokinetics of chimeric mAb variants in ferrets

**Figure 6:**
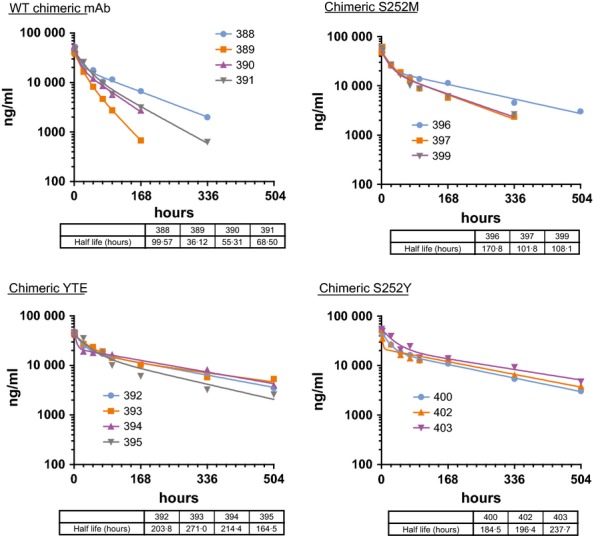
First dose PK. mAb concentrations in the plasma from individual ferrets given the wild-type (WT) chimeric mAb and the three variants are shown. Terminal half-life values based on a nonlinear fit of the data points are given in the table below each graph.

The pharmacokinetics of the WT chimeric mAb and the S252M, S252Y, and YTE variants were evaluated in ferrets. Male ferrets were intravenously injected with a 2-mg/kg dose of mAb. Blood samples were then collected 2, 6, 12, 24, 48, 72, 96, 168, 336, and 504 hours after injection, and plasma prepared from each sample. Each ferret was given a second injection of test article after the last sampling and blood samples collected at the same time points relative to the second injection. Concentrations of human mAb in the plasma samples were measured using the previously described electrochemiluminescent assay. The average terminal half-lives of the S252M, S252Y, and YTE variants were 127 ± 38, 206 ± 44, and 213 ± 28 hours, respectively. All three showed an extended half-life compared with WT chimeric mAb with a half-life of 65 ± 27 hours. Figure6 shows the mAb concentrations determined for individual ferrets after the first injection. Results from two of the ferrets, one in the in the S252M group and another in the S252Y group, are not shown because technical difficulties with the vasculature access port prevented sample collection. mAb concentrations were unusually variable in plasma samples from four ferrets given WT chimeric mAb, with calculated half-life values ranging from 36 to 100 hours. The mAb concentrations in the plasma samples from ferrets given the three variants were more consistent.

**Figure 7:**
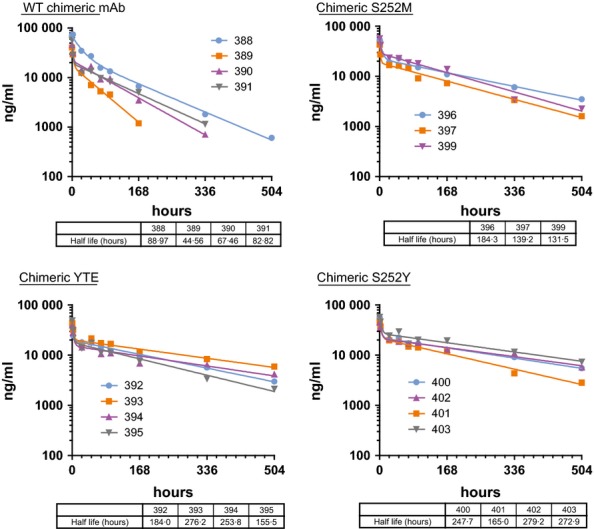
Second dose PK. mAb concentrations in the plasma from individual ferrets given the wild-type (WT) chimeric mAb and the three Fc are shown. Terminal half-life values based on a nonlinear fit of the data points are given in the table below each graph.

Figure7 shows mAb concentrations in plasma samples taken after the second dose, and Table1 gives a comparison of half-lives determined from samples taken after the first and second doses. Overall, the half-lives were similar to what was observed for the first injection. Importantly, what was not seen in any of the ferrets was an abrupt decrease in chimeric mAb concentration that would be suggestive of an ADA response.

**Table 1:** Comparison of half-lives after the first and second doses. The half-lives of the human/ferret chimeric mAbs were individually calculated based on samples taken after the first or second dose. A decrease in half-life after the second dose might indicate an immune response. The percent difference between the first and second dose is given with a positive number indicating an increased half-life after the second dose and a negative number indicating a decreased half-life

Test article	Animal number	1st dose *T*_1/2_ (hours)	2nd dose *T*_1/2_ (hours)	% Difference
Chimeric mAb	388	100	89	−11
	389	36	45	+23
	390	55	67	+22
	391	69	83	+21
Chimeric YTE	392	204	184	−10
	393	271	276	+2
	394	214	254	+18
	395	165	156	−5
Chimeric S252M	396	171	184	+8
	397	102	139	+37
	398	n.d.	n.d.	n.d.
	399	108	132	+22
Chimeric S252Y	400	185	248	+34
	401	n.d.	165	n.d.
	402	196	279	+42
	403	238	273	+15

The notation “n.d.” indicates that data were not available due to technical issues.

**Figure 8:**
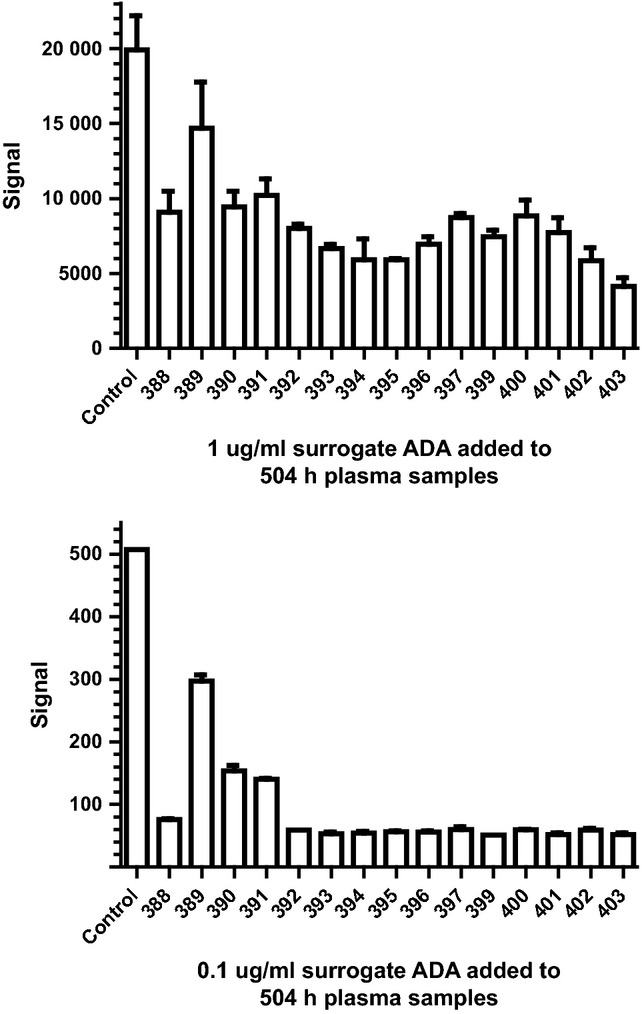
Inhibition of immune response. The ability of ferret plasma samples taken at the 504 hour time point to inhibit the detection of a surrogate antidrug antibody was measured.

In an attempt to confirm that detectable ADAs were not generated against the chimeric mAbs, a bridging assay similar to the one used with the human mAbs was used to analyze plasma samples. No ADAs were detected in the final plasma samples, taken 504 hours after the second dose (data not shown). A shortcoming of this assay format was the potential for false-negative results due to residual test article in the plasma samples. For this reason, a control anti-idiotype mAb against the variable regions of the chimeric mAbs was used as a surrogate ADA to determine whether residual chimeric mAb in the plasma would interfere with the bridging assay. The anti-idiotype mAb (representing surrogate ADA) was spiked into the 504-hour plasma samples, and then those samples tested in the same assay. Detection of the anti-idiotype mAb at a concentration of 1 μg/ml was partially inhibited in the 504-hour plasma samples, and when the concentration was reduced to 0·1 μg/ml, its detection was completely inhibited in most of the plasma samples (Figure8). Detection was not inhibited when it was added to control ferret plasma. These results suggested that residual chimeric mAb still present in the plasma samples could interfere with detection of ADA. Consistent with this notion, the plasma samples with the lowest concentration of residual chimeric mAb showed the least inhibition when assaying for the anti-idiotype mAb. Therefore, while the consistent rate of clearance over time observed for the human/ferret chimeric mAbs suggests a lack of ADA response, it was unable to be confirmed through direct testing.

## Discussion

The wish to evaluate antibodies in ferrets without having to dose at frequent intervals and without having ferret ADA immune responses interfere with antibody efficacy prompted our effort to test whether these issues could be minimized by redesigning the test antibodies. As we were not aware of published reports that explicitly described the pharmacokinetics of human mAbs in ferrets, we first determined the half-life of a human mAb. For this, a human IgG mAb not thought to bind endogenous ferret antigens (the mAb is specific for the F glycoprotein of RSV) was used as test antibody in order to avoid any antigen-mediated effects on antibody pharmacokinetics. When the PK of the original human IgG1 mAb was studied in ferrets, we observed a half-life of 31 ± 10 hours, longer than the 14 hours we estimated from the published work of Bossart *et al*.,^4^ but still much shorter than what has typically been observed in other common models such as mice and cynomolgus monkeys. The introduction of two amino acid substitutions in the human IgG1 Fc, H433L, and N434S, extended its half-life to 72 ± 21 hours, a 2·3-fold improvement over the original 31 hours. Although this met our initial goal of a 3-day half-life, some ferrets showed indications of an ADA response. The presence of ADAs was confirmed in two plasma samples, and a third showed a rapid drop in plasma mAb concentrations indicative of an immune response. This sample had a sixfold higher plasma concentration of residual human mAb, which may have blocked detection of ADAs in our assay. Antibody immune responses against heterologous test antibodies in model species are a common issue, and the incidence increases when more than one dose is given. Because ADAs usually develop 7–10 days after exposure, studies which extend beyond this point risk the effects of ADA-mediated clearance on efficacy. Avoiding ADA responses would be more likely if the antibody test articles contained ferret constant regions. Therefore, we cloned the ferret HC and LC constant regions and used them to make human/ferret chimeric antibodies. Having seen that substitutions in the human Fc could increase half-life in the ferret, several substitutions which had been shown to increase the half-life of human antibodies were introduced into the ferret Fc. The chimeric mAb and variants were screened in ferret FcRn binding assays. Because the sequence of ferret FcRn was only partially known, it also had to first be cloned from ferret tissues. It should be noted that while FcRn binding at pH 6 has been used to compare antibodies engineered for increased half-life, many antibodies which display tighter binding at pH 6 also bind more tightly at higher pH and are not efficiently released. Therefore, FcRn binding at pH 6 can be useful as an initial screening tool, but must be confirmed by *in vivo* testing. FcRn binding analyzed by an AlphaScreen-based assay revealed the following rank order of affinities for the variants: S252Y=YTE>S252M>N434S>WT. The finding that the serine at position 252 naturally found in the ferret antibody sequence results in decreased binding to ferret FcRn compared with the methionine found at this position in humans is interesting and gives support to the idea that the intrinsic clearance of endogenous antibodies in ferrets is more rapid than in other species. The WT chimeric mAb and three variants, S252M, S252Y, and YTE, were then included in a ferret PK study. The half-lives of the chimeric mAbs *in vivo* were consistent with the FcRn binding assays, showing a rank order of half-lives of S252Y=YTE>S252M>WT. The strongest FcRn binders S252Y and YTE had half-lives of ∼9 days. However, perhaps the most important observation from this study was the lack of precipitous drops in antibody plasma concentration which would indicate strong ADA responses. Others have described dramatic decreases in injected antibody concentrations as pharmacokinetic indications of an ADA response.^14^ Preferably, this could have been supported by the direct detection of ADAs; however, residual test article in the plasma samples partially inhibited detection. Therefore, while the observations presented here make a strong case that chimeric human/ferret antibodies are less immunogenic in ferrets, the possibility exists that weak immune responses went undetected. Future studies might include a wash-out period to allow injected antibody to be fully cleared.

Overall, these results show that a chimeric mAb with ferret constant regions can be used to minimize immunogenicity in ferrets and can be engineered for a long half-life. The chimeric mAbs were properly assembled, were biophysically well-behaved, and retained full antigen binding. It should be straightforward to prepare such chimeric antibodies from any antibody variable regions and thereby gain the advantages of lower dosage and less frequent dosing, as well as reduced variability between animals, especially in long-term multidose studies.
